# A study on the effect of posture-recognition-based mirror visual feedback therapy on balance function and activities of daily living in patients with poststroke neglect

**DOI:** 10.1097/MD.0000000000050072

**Published:** 2026-08-14

**Authors:** Juan Jin, Hongji An, Juanxiu Cui, Long Piao

**Affiliations:** a Department of Rehabilitation Medicine, The Affiliated Hospital of Yanbian University (Yanbian Hospital), Yanji City, Yanbian Korean Autonomous Prefecture, Jilin Province, China.

**Keywords:** activities of daily living, balance function, mirror visual feedback, posture recognition, stroke, unilateral spatial neglect

## Abstract

This study aimed to investigate the effects of posture-recognition-based mirror visual feedback therapy (MVFT) on the degree of neglect, balance function, and activities of daily living in patients with poststroke neglect. A retrospective cohort study design was employed. Patients with poststroke neglect admitted to the Department of Rehabilitation Medicine of our hospital from May 2023 to May 2025 were selected and divided into a feedback therapy group and a conventional rehabilitation group based on whether they received posture-recognition-based MVFT. Propensity score matching was used to perform a 1:1 match between the 2 groups, ultimately including 63 patients in each group. Scores from the Line Cancellation Test, the Catherine Bergego Scale (CBS), the Berg Balance Scale (BBS), and the modified Barthel Index (MBI) were collected before treatment and after 4 weeks of treatment. Differences in treatment effects between the 2 groups were compared, and the correlation between the improvement in neglect and functional recovery was analyzed. After matching, baseline data were balanced and comparable between the 2 groups (*P* > .05). After treatment, the feedback therapy group showed significantly greater improvements than the conventional rehabilitation group in the Line Cancellation Test (8.44 ± 3.21 vs 5.67 ± 2.98, *P* = .003), CBS score (7.22 ± 3.01 vs 5.11 ± 2.87, *P* = .005), BBS score (17.22 ± 5.67 vs 12.14 ± 5.23, *P* = .001), and MBI score (26.78 ± 8.45 vs 18.34 ± 7.89, *P* < .001). Correlation analysis revealed that the improvement in CBS score was significantly positively correlated with the improvement in BBS score (*r* = 0.512, *P* < .001) and the improvement in MBI score (*r* = 0.548, *P* < .001). Subgroup analysis indicated that the therapy was effective for patients with lesions on either side (*P* < .05) and that its efficacy was not affected by the side of the lesion (*P* > .05). Posture-recognition-based MVFT can effectively reduce neglect symptoms, improve balance function, and enhance activities of daily living in patients with poststroke neglect. The alleviation of neglect is closely related to functional recovery. This therapy demonstrates good applicability to patients with lesions on either side and is worthy of clinical promotion.

## 1. Introduction

Stroke is one of the leading causes of adult disability, with approximately 25% to 30% of stroke survivors experiencing unilateral spatial neglect (USN), characterized by an inability to perceive, respond to, or orient toward stimuli on the side contralateral to the brain lesion.^[1,2]^ Neglect syndrome not only severely impacts patients’ visual perception and motor output but is also closely associated with an increased risk of falls, delayed rehabilitation progress, and decreased capacity for activities of daily living. It is considered a negative predictor of functional recovery after stroke.^[3,4]^ Therefore, effectively ameliorating neglect symptoms and promoting the recovery of balance function and activities of daily living have consistently been focal points of research in the field of rehabilitation medicine.

Mirror visual feedback therapy (MVFT) is one of the commonly used interventions for neglect syndrome. Its basic principle involves reflecting the image of the moving unaffected limb through a plane mirror, creating a visual illusion of normal movement in the affected limb for the patient. This is thought to activate the mirror neuron system in the affected cerebral hemisphere, facilitating the reorganization of the damaged spatial attention network.^[5,6]^ Previous studies have confirmed that conventional mirror therapy can improve neglect symptoms to a certain extent. However, its efficacy shows individual variability and is limited by the monotonous nature of the training format – patients can only passively observe the mirror image. The lack of precise capture and real-time feedback on the actual movement posture of the affected limb often leads to compensatory movements, making it difficult to maximize the training effect.^[7,8]^

In recent years, with the development of artificial intelligence and computer vision technology, posture-recognition-based MVFT has emerged. This technology uses depth cameras to capture the movement trajectory of the patient’s unaffected limb in real time. Computer algorithms are then employed to generate a dynamic mirror image of the affected limb, integrating visual search tasks targeting the neglected side and specific posture simulation training.^[9]^ Compared with conventional mirror therapy, posture-recognition technology can provide more precise and enriched multisensory integration input. It not only strengthens visual-motor feedback but also corrects abnormal postures in real time, potentially activating the inhibited spatial attention network on the affected side more effectively.^[10]^ Preliminary studies suggest that this technology shows promise in improving motor function after stroke^[11]^; however, sufficient clinical evidence regarding its effects on balance function and activities of daily living in patients with neglect syndrome is still lacking, particularly in head-to-head comparisons with conventional therapy.

Therefore, this study employed a retrospective cohort study design, using propensity score matching to balance confounding factors. We compared the effects of posture-recognition-based MVFT versus conventional rehabilitation training on the degree of neglect, balance function, and activities of daily living in patients with poststroke neglect. Furthermore, we explored the correlation between the improvement in neglect and functional recovery, aiming to provide evidence-based support for optimizing clinical rehabilitation protocols.

## 2. Methods

### 2.1. Study design

This study was approved by the Ethics Committee of Yanbian University Affiliated Hospital. It employed a retrospective cohort study design. Using a convenience sampling method, hospitalized stroke patients admitted to the Department of Rehabilitation Medicine of our hospital from May 2023 to May 2025 were selected as the study population. Case data were rigorously screened according to preestablished inclusion and exclusion criteria. Based on whether patients received posture-recognition-based MVFT during their hospitalization, the enrolled patients were divided into 2 groups: those who received this therapy were assigned to the feedback therapy group, and those who did not receive this therapy and only underwent conventional rehabilitation training were assigned to the conventional rehabilitation group.

Because this was a retrospective cohort study based on routinely collected clinical data, no prospective sample size calculation was performed. Instead, all eligible patients admitted between May 2023 and May 2025 who satisfied the predefined inclusion and exclusion criteria were consecutively included. After propensity score matching, 63 matched pairs were available for the final analysis.

To control for the influence of confounding factors such as age, disease duration, and baseline severity on the study results, propensity score matching was used to perform a 1:1 match between the 2 groups, with a caliper value set at 0.02. Ultimately, 63 patients were included in each group, ensuring comparability of baseline data between the 2 groups.

### 2.2. Inclusion and exclusion criteria

Inclusion criteria were as follows: first-ever stroke (including ischemic or hemorrhagic) confirmed by computed tomography or magnetic resonance imaging; disease duration within 6 months; presence of USN, with positive results on the Line Cancellation Test or Letter Cancellation Test; stable vital signs, being conscious, and being able to follow commands to complete rehabilitation training; and complete clinical record data.

Exclusion criteria were as follows: comorbid severe cognitive impairment (e.g., Mini-Mental State Examination score below the cutoff value) or aphasia affecting comprehension; severe visual impairment or visual field defects (unless the medical record explicitly ruled out their impact on training); previous history of mental illness or severe bone and joint diseases affecting balance function; and missing data for key assessment indicators (e.g., Berg Balance Scale [BBS] and modified Barthel Index [MBI]) in the clinical medical records.

### 2.3. Grouping and treatment methods

#### 2.3.1. Grouping method

The observation group consisted of patients who received posture-recognition-based MVFT in addition to conventional rehabilitation treatment. The control group consisted of patients hospitalized during the same period who received only conventional mirror therapy or conventional occupational therapy training in addition to conventional rehabilitation treatment.

#### 2.3.2. Treatment methods

The interventions involved in this study were extracted and verified based on the rehabilitation treatment records documented in the medical record system. Both groups of patients received conventional basic treatment from the Department of Rehabilitation Medicine, including physical therapy (e.g., comprehensive training for hemiplegic limbs and balance function training) and occupational therapy (e.g., activities of daily living training), with a total daily treatment duration of approximately 2 hours. On this basis, the 2 groups received the following specific interventions, respectively:

Patients in the observation group underwent training using posture-recognition-based MVFT. This therapy utilized depth cameras to capture the movement trajectory of the patient’s unaffected upper or lower limb. Computer algorithms generated a real-time dynamic mirror image of the affected limb, which was projected onto a screen. The training content included not only bilateral symmetrical movements but also integrated visual search tasks targeting the neglected side and specific posture simulation tasks, aiming to strengthen spatial attention on the affected side through visuomotor integration. According to the medical records, each session of this treatment lasted 30 minutes, was administered once daily, for 5 consecutive days per week, over a total period of 4 weeks.

Patients in the control group received conventional mirror therapy. The specific procedure was as follows: the patient sat in front of a treatment table, with a vertically placed mirror positioned along the body’s midsagittal plane. The unaffected upper or lower limb was placed on the reflective side of the mirror, while the affected limb was hidden behind the mirror. The patient focused on the image of the unaffected limb in the mirror to create the visual illusion of movement in the affected limb, performing exercises such as joint flexion, extension, and rotation. For patients unable to perform conventional mirror therapy due to their condition, conventional occupational therapy was provided, consisting of functional training for the affected upper limb and sensory stimulation. The duration of each session, treatment frequency, and total course for the control group were consistent with those of the observation group, that is, 30 minutes per session, once daily, 5 days per week, for 4 weeks.

### 2.4. Data collection

This study retrospectively collected patients’ clinical data through the hospital medical record system. All data were independently extracted and cross-checked by 2 trained researchers. The collected information included demographic characteristics, disease-related characteristics, basic medication status, disease severity, cognitive function, emotional state, comorbidity burden, and scores on various assessment scales. The specific collection content and time points are as follows:

#### 2.4.1. Collection of demographic and disease-related characteristic data

Demographic characteristics and disease-related data were collected upon patient admission. Demographic characteristics included age and sex. Disease-related characteristics included the nature of the lesion (ischemic/hemorrhagic), the side of the lesion (left/right), and the disease duration (days from onset to admission). The above information was sourced from admission records and imaging reports.

#### 2.4.2. Collection of basic medication status data

Basic medication status during hospitalization was collected. According to medical order records, the categories of medications used by patients were documented, including antiplatelet/anticoagulant drugs (e.g., aspirin, clopidogrel, and warfarin), antihypertensive drugs (e.g., angiotensin-converting enzyme inhibitors/angiotensin II receptor blockers, calcium channel blockers, and beta-blockers), and lipid-lowering drugs (e.g., statins and ezetimibe). Data were recorded as the number and percentage of patients using each category.

#### 2.4.3. Collection of disease severity, cognitive function, and emotional state data

Assessments of disease severity, cognitive function, and emotional state were completed within 3 days of patient admission. Disease severity was evaluated using the National Institutes of Health Stroke Scale, where higher scores indicate more severe neurological deficits. Cognitive function was assessed using the Montreal Cognitive Assessment, where lower scores indicate more significant cognitive impairment. Emotional state was evaluated using the Patient Health Questionnaire-9, where higher scores indicate more severe depressive symptoms. The above assessment results were extracted from the rehabilitation evaluation records in the medical record system.

#### 2.4.4. Collection of comorbidity burden data

Information regarding comorbidities was collected upon patient admission and calculated according to the Charlson Comorbidity Index scoring criteria. This index comprehensively considers the impact of various comorbidities (such as myocardial infarction, heart failure, diabetes, and chronic pulmonary disease) on prognosis, with higher scores indicating a greater comorbidity burden. The data were sourced from the past medical history and comorbidity diagnoses documented in the admission records.

#### 2.4.5. Collection of neglect severity assessment data

Neglect severity assessment data were collected before treatment (within 3 days of admission) and after 4 weeks of treatment (at the end of the intervention). The Line Cancellation Test was used to assess the degree of USN. During the test, patients were required to cancel lines randomly distributed on an A4 sheet of paper; a higher number of omissions indicated more severe neglect. Simultaneously, the Catherine Bergego Scale (CBS) was used to assess the severity of neglect behaviors in daily life. This scale evaluates patients’ performance in 10 daily activities, such as eating, dressing, and grooming, with scores ranging from 0 to 30. Higher scores indicate more pronounced neglect behaviors in daily life. The above assessment results were extracted from the rehabilitation treatment records.

#### 2.4.6. Collection of balance function assessment data

Balance function assessment data were collected before treatment (within 3 days of admission) and after 4 weeks of treatment (at the end of the intervention). The BBS was used to assess patients’ balance function. This scale comprises 14 items, including sitting unsupported, transfers, and standing on 1 leg, with a total score of 56. Higher scores indicate better balance function. The assessment results were sourced from the evaluation records of the rehabilitation therapists.

#### 2.4.7. Collection of activities of daily living assessment data

Activities of daily living assessment data were collected before treatment (within 3 days of admission) and after 4 weeks of treatment (at the end of the intervention). The MBI was used to assess patients’ capacity for activities of daily living. This scale comprises 10 items, including feeding, dressing, toileting, transfers, and walking, with a total score of 100. Higher scores indicate greater independence in activities of daily living. The assessment results were sourced from occupational therapy records or nursing evaluation records.

#### 2.4.8. Data quality control

To ensure the accuracy and completeness of the data, all data were independently extracted by 2 researchers and entered into an Excel database. After entry, cross-checking was performed. For any discrepancies in the data, a third researcher reviewed the information, and consensus was reached through discussion. Cases with missing or incomplete records for key assessment indicators were excluded.

### 2.5. Statistical analysis

SPSS 26.0 (IBM Corp.) statistical software was used for data analysis. Measurement data were expressed as mean ± standard deviation (*x̄* ± *s*), and count data were expressed as numbers and percentages (%). For comparisons of baseline data between groups, an independent samples *t* test was used for measurement data, and the chi-square test was used for count data. Comparisons of scores within each group before and after treatment were performed using a paired samples *t* test. Comparisons of the improvement values (score changes) between the 2 groups before and after treatment were performed using an independent samples *t* test. The correlation between the improvement in neglect severity and functional recovery was analyzed using Pearson correlation analysis. In subgroup analysis, comparisons of therapeutic effects between the 2 groups within the same lesion side, as well as comparisons between different lesion sides within the same treatment group, were both performed using independent samples *t* tests. A *P*-value < .05 was considered statistically significant. Propensity score matching was performed to minimize selection bias and to balance measured baseline covariates between groups. However, as with all retrospective observational studies, residual confounding from unmeasured variables cannot be completely excluded.

## 3. Results

### 3.1. Comparison of baseline data between the 2 groups

After propensity score matching, 63 patients were included in each group. There were no statistically significant differences between the 2 groups in terms of age, sex, nature of the lesion, side of the lesion, disease duration, basic medication (antiplatelet/anticoagulant, antihypertensive, and lipid-lowering drugs), disease severity (National Institutes of Health Stroke Scale score), cognitive function (Montreal Cognitive Assessment score), emotional state (Patient Health Questionnaire-9 score), comorbidity burden (Charlson Comorbidity Index), and pretreatment scores on the Line Cancellation Test, CBS, BBS, and MBI (*P* > .05), indicating that the baseline data were balanced and comparable between the 2 groups (Table 1).

**Table 1:** Comparison of baseline data between the 2 groups after propensity score matching.

Indicator	Feedback therapy group (n = 63)	Conventional rehabilitation group (n = 63)	Statistical value	*P*-value
Demographic characteristics				
Age (yr, *x̄* ± *s*)	58.34 ± 9.87	59.12 ± 10.23	*t* = 0.435	.665
Sex (n [%])			χ^2^ = 0.129	.72
Male	38 (60.3)	36 (57.1)		
Female	25 (39.7)	27 (42.9)		
Disease-related characteristics				
Nature of lesion (n [%])			χ^2^ = 0.032	.858
Ischemic	41 (65.1)	42 (66.7)		
Hemorrhagic	22 (34.9)	21 (33.3)		
Side of lesion (n [%])			χ^2^ = 0.292	.589
Left	34 (54.0)	31 (49.2)		
Right	29 (46.0)	32 (50.8)		
Disease duration (d, *x̄* ± *s*)	68.45 ± 25.67	71.23 ± 27.89	*t* = 0.583	.561
Basic medication				
Antiplatelet/anticoagulant drugs (n [%])	58 (92.1)	59 (93.7)	χ^2^ = 0.121	.724
Antihypertensive drugs (n [%])	48 (76.2)	50 (79.4)	χ^2^ = 0.185	.667
Lipid-lowering drugs (n [%])	52 (82.5)	54 (85.7)	χ^2^ = 0.242	.623
Disease severity				
NIHSS score (points, *x̄* ± *s*)	8.56 ± 3.21	8.98 ± 3.45	*t* = 0.712	.478
Cognitive function				
MoCA score (points, *x̄* ± *s*)	21.34 ± 3.45	20.98 ± 3.67	*t* = 0.567	.572
Emotional state				
PHQ-9 score (points, *x̄* ± *s*)	6.78 ± 3.12	7.01 ± 3.45	*t* = 0.389	.698
Comorbidity burden				
Charlson Comorbidity Index (points, *x̄* ± *s*)	2.34 ± 1.12	2.45 ± 1.23	*t* = 0.523	.602
Baseline assessment indicators				
Line Cancellation Test (points, *x̄* ± *s*)	18.67 ± 5.43	19.12 ± 5.87	*t* = 0.446	.657
CBS score (points, *x̄* ± *s*)	15.89 ± 4.56	16.34 ± 4.98	*t* = 0.527	.599
BBS score (points, *x̄* ± *s*)	28.45 ± 7.89	27.98 ± 8.23	*t* = 0.328	.744
MBI score (points, *x̄* ± *s*)	45.67 ± 12.34	44.89 ± 13.01	*t* = 0.346	.73

BBS = Berg Balance Scale, CBS = Catherine Bergego Scale, MBI = modified Barthel Index, MoCA = Montreal Cognitive Assessment, NIHSS = National Institutes of Health Stroke Scale, PHQ-9 = Patient Health Questionnaire-9.

### 3.2. Comparison of Line Cancellation Test scores between the 2 groups before and after treatment

The Line Cancellation Test is a commonly used objective indicator for assessing the degree of USN. Before treatment, there was no statistically significant difference in Line Cancellation Test scores between the 2 groups (*P* > .05). After treatment, scores in both groups decreased significantly compared with those before treatment, with statistically significant differences within each group (*P* < .001). The improvement in scores before and after treatment was (8.44 ± 3.21) points in the feedback therapy group and (5.67 ± 2.98) points in the conventional rehabilitation group, with a statistically significant difference between the groups (*t* = 3.012, *P* = .003; Table 2).

**Table 2:** Comparison of Line Cancellation Test scores between the 2 groups before and after treatment (points, *x̄* ± *s*).

Group	n	Before treatment	After treatment	Improvement value*	Intra-group *P*-value†	Inter-group *P*-value‡
Feedback therapy group	63	18.67 ± 5.43	10.23 ± 4.12	8.44 ± 3.21	<.001	.003
Conventional rehabilitation group	63	19.12 ± 5.87	13.45 ± 4.89	5.67 ± 2.98	<.001	

* Improvement value = Pretreatment score − Posttreatment score.

† Intra-group comparison using paired samples *t* test.

‡ Inter-group comparison of improvement values using independent samples *t* test, *t* = 3.012.

### 3.3. Comparison of CBS scores between the 2 groups before and after treatment

The CBS is used to assess the severity of neglect behaviors in daily life. Before treatment, there was no statistically significant difference in CBS scores between the 2 groups (*P* > .05). After treatment, CBS scores in both groups decreased significantly compared with before treatment (*P* < .001), and the improvement value in the feedback therapy group was (7.22 ± 3.01) points, which was significantly higher than (5.11 ± 2.87) points in the conventional rehabilitation group (*t* = 2.867, *P* = .005), suggesting that the feedback therapy group achieved better effects in improving neglect behaviors in daily life (Table 3).

**Table 3:** Comparison of Catherine Bergego Scale scores between the 2 groups before and after treatment (points, *x̄* ± *s*).

Group	n	Before treatment	After treatment	Improvement value*	Intra-group *P*-value†	Inter-group *P*-value‡
Feedback therapy group	63	15.89 ± 4.56	8.67 ± 3.45	7.22 ± 3.01	<.001	.005
Conventional rehabilitation group	63	16.34 ± 4.98	11.23 ± 4.12	5.11 ± 2.87	<.001	

CBS = Catherine Bergego Scale.

* Improvement value = Pretreatment score − Posttreatment score.

† Intra-group comparison using paired samples *t* test.

‡ Inter-group comparison of improvement values using independent samples *t* test, *t* = 2.867. Lower CBS scores indicate fewer neglect behaviors in daily life.

### 3.4. Comparison of BBS scores between the 2 groups before and after treatment

The BBS is a commonly used objective indicator for assessing balance function. Before treatment, BBS scores were at a similar level between the 2 groups (*P* > .05). After 4 weeks of intervention, balance ability improved significantly in both groups (*P* < .001), but the degree of improvement was more pronounced in the feedback therapy group, with an improvement value of (17.22 ± 5.67) points, which was significantly higher than (12.14 ± 5.23) points in the conventional rehabilitation group (*t* = 3.456, *P* = .001; Table 4).

**Table 4:** Comparison of Berg Balance Scale scores between the 2 groups before and after treatment (points, *x̄* ± *s*).

Group	n	Before treatment	After treatment	Improvement value*	Intra-group *P*-value†	Inter-group *P*-value‡
Feedback therapy group	63	28.45 ± 7.89	45.67 ± 8.34	17.22 ± 5.67	<.001	.001
Conventional rehabilitation group	63	27.98 ± 8.23	40.12 ± 8.89	12.14 ± 5.23	<.001	

* Improvement value = Posttreatment score − Pretreatment score.

† Intra-group comparison using paired samples *t* test.

‡ Inter-group comparison of improvement values using independent samples *t* test, *t* = 3.456.

### 3.5. Comparison of MBI scores between the 2 groups before and after treatment

Regarding the capacity for activities of daily living, there was no significant difference in MBI scores between the 2 groups before treatment (*P* > .05). After 4 weeks of rehabilitation training, the activities of daily living of patients in both groups improved significantly (*P* < .001). However, the degree of improvement in the feedback therapy group was more remarkable, with an average increase of (26.78 ± 8.45) points, substantially surpassing the (18.34 ± 7.89) points in the conventional rehabilitation group. The difference between the groups was statistically significant (*t* = 4.234, *P* < .001; Table 5).

**Table 5:** Comparison of modified Barthel Index scores between the 2 groups before and after treatment (points, *x̄* ± *s*).

Group	n	Before treatment	After treatment	Improvement value*	Intra-group *P*-value†	Inter-group *P*-value‡
Feedback therapy group	63	45.67 ± 12.34	72.45 ± 13.56	26.78 ± 8.45	<.001	<.001
Conventional rehabilitation group	63	44.89 ± 13.01	63.23 ± 14.12	18.34 ± 7.89	<.001	

* Improvement value = Posttreatment score − Pretreatment score.

† Intra-group comparison using paired samples *t* test.

‡ Inter-group comparison of improvement values using independent samples *t* test, *t* = 4.234.

### 3.6. Correlation analysis between neglect improvement and functional recovery

To further explore the relationship between the alleviation of neglect symptoms and functional recovery, a correlation analysis was conducted on the improvement values of various indicators before and after treatment for all patients. The results showed that the improvement in CBS scores was moderately positively correlated with the improvements in BBS scores and MBI scores. Similarly, the improvement in Line Cancellation Test scores was also significantly positively correlated with the improvements in BBS scores and MBI scores (Table 6).

**Table 6:** Correlation analysis between improvement in neglect and improvement in balance function and activities of daily living.

Indicator	Statistical value	CBS score improvement	Line Cancellation Test score improvement
BBS score improvement	*r*-value	0.512	0.467
	*P*-value	<.001	<.001
MBI score improvement	*r*-value	0.548	0.489
	*P*-value	<.001	<.001

Pearson correlation analysis was used; all *P* < .001, indicating statistically significant correlations.

BBS = Berg Balance Scale, CBS = Catherine Bergego Scale, MBI = modified Barthel Index.

### 3.7. Subgroup analysis of improvement values after treatment in patients with different lesion sides

To investigate whether the side of the lesion influences treatment efficacy, this study further conducted a subgroup analysis based on the side of neglect. The analysis revealed that, for both patients with left-sided neglect and those with right-sided neglect, the improvements in BBS and MBI scores in the feedback therapy group were significantly higher than those in the conventional rehabilitation group (*P* < .05). However, within the same treatment group, there were no statistically significant differences in the improvement values between patients with left-sided neglect and those with right-sided neglect (*P* > .05; Table 7).

**Table 7:** Inter-group comparison of improvement values after treatment in patients with different lesion sides (points, *x̄* ± *s*).

Indicator	Lesion side	n (feedback/conventional)	Feedback therapy group improvement value	Conventional rehabilitation group improvement value	Inter-group *P*-value*
BBS improvement	Left-sided neglect	34/31	18.23 ± 5.67	12.45 ± 5.12	.002
	Right-sided neglect	29/32	16.01 ± 5.23	11.89 ± 5.34	.018
	Inter-stratum *P*-value†		.124	.678	
MBI improvement	Left-sided neglect	34/31	27.89 ± 8.56	18.67 ± 7.89	<.001
	Right-sided neglect	29/32	25.45 ± 8.12	18.01 ± 7.67	.003
	Inter-stratum *P*-value†		.256	.734	

BBS = Berg Balance Scale, MBI = modified Barthel Index.

* Comparison of improvement values between the feedback therapy group and the conventional rehabilitation group within the same lesion side.

† Comparison of improvement values between patients with left-sided neglect and those with right-sided neglect within the same treatment group.

## 4. Discussion

The results of this study indicate that posture-recognition-based MVFT is significantly superior to conventional rehabilitation training in improving neglect symptoms, balance function, and activities of daily living in patients with poststroke neglect. Furthermore, the reduction in neglect severity was positively correlated with functional recovery, and the therapeutic efficacy was not influenced by the side of the lesion.

Regarding the improvement in neglect symptoms, the feedback therapy group demonstrated significantly greater improvements in both the Line Cancellation Test and CBS scores compared with the conventional rehabilitation group. This advantage may be associated with the multisensory integration training provided by posture-recognition technology.^[12]^ Although conventional mirror therapy can activate the mirror neuron system through visual illusion, it lacks sensory feedback regarding the actual movement state of the affected limb.^[13]^ In contrast, posture-recognition-based technology captures the movements of the unaffected limb in real time using depth cameras, generates a precise mirror image of the affected limb, and integrates this with visual search tasks targeting the neglected side. This provides patients with multiple inputs encompassing vision, proprioception, and motor execution. Such multisensory integration can more potently activate the brain’s attention network, particularly the parietal cortex regions involved in spatial attention, thereby facilitating the reorientation of attention toward the neglected side of space.^[14]^ The posture simulation tasks incorporated into the training require patients to actively adjust their body posture, and this active engagement may further strengthen neuroplastic changes. One possible explanation is that posture-recognition-based MVFT may enhance multisensory integration, thereby facilitating activation of neural networks involved in spatial attention. However, the present study did not include neuroimaging or neurophysiological assessments, and this proposed mechanism remains speculative.

In terms of balance function, the improvement in BBS scores in the feedback therapy group was significantly superior to that in the conventional rehabilitation group, suggesting that this therapy has a better promoting effect on balance ability. The maintenance of balance function relies on multisensory integration and the precise regulation of the central nervous system.^[15]^ Due to their perceptual deficits toward the affected side of space, patients with neglect syndrome often fail to perceive environmental changes on the affected side and their own postural deviations in a timely manner, leading to a shift in the center of gravity towards the unaffected side and an increased risk of falls. In this study, as neglect symptoms alleviated, patients’ attentional ability toward the affected side of space improved, enabling them to perceive visual information from the affected side more keenly and adjust their posture accordingly.^[16]^ Concurrently, the posture simulation tasks during training may have directly strengthened the central nervous system’s regulatory capacity over balance. The correlation analysis revealed a positive correlation between neglect improvement and balance function enhancement, further supporting the inference that the alleviation of neglect symptoms is an important contributing factor to the improvement of balance function.

Regarding the capacity for activities of daily living, the improvement in MBI scores in the feedback therapy group was significantly higher than that in the conventional rehabilitation group, indicating that this therapy can more effectively enhance patients’ practical living abilities. Activities of daily living require the coordinated integration of spatial attention, postural control, and motor execution.^[17]^ Patients with neglect syndrome often experience limitations in activities such as dressing and eating due to focusing only on the unaffected side of space. In this study, with the improvement in neglect symptoms, patients were able to distribute their attention more evenly to both sides of space, reducing neglect behaviors in daily activities. Furthermore, the enhancement of balance function laid a foundation for patients to perform daily activities more safely. The correlation analysis showed a moderate positive correlation between neglect improvement and MBI improvement, suggesting that the alleviation of neglect symptoms can translate into enhanced practical living abilities. However, it also indicates that the recovery of activities of daily living is influenced by multiple factors, including motor function, muscle strength, and psychological state.^[18]^

Subgroup analysis revealed that for both patients with left-sided neglect and those with right-sided neglect, the therapeutic effects in the feedback therapy group were significantly superior to those in the conventional rehabilitation group. Furthermore, within the same treatment group, there were no significant differences in improvement values between patients with lesions on different sides. This indicates that the therapy has good applicability to patients with neglect irrespective of the lesion side. From a neuroanatomical perspective, left-sided neglect is typically associated with right hemisphere damage, while right-sided neglect is associated with left hemisphere damage, with differences in the impairment patterns of attentional networks between the 2 groups.^[19,20]^ However, the consistency of the therapeutic effects observed in this study suggests that this therapy may exert its intervention effects on different types of neglect by activating the mirror neuron systems and attentional networks in both cerebral hemispheres, demonstrating strong generalizability.^[21]^

This study has certain limitations. First, although propensity score matching substantially improved baseline comparability, this retrospective study remains susceptible to residual selection bias and unmeasured confounding. Therefore, causal inference regarding the superiority of posture-recognition-based MVFT should be interpreted cautiously. Future multicenter prospective randomized controlled trials are warranted to validate these findings. Second, the sample size was relatively limited, potentially resulting in insufficient statistical power for the subgroup analyses. Third, the intervention period was only 4 weeks, and long-term follow-up data are lacking. Fourth, differences in treatment response among different subtypes of neglect were not explored in depth. Future prospective randomized controlled trials with extended follow-up periods, combined with neuroimaging techniques, are warranted to investigate the mechanisms of action of this therapy.

Our findings suggest that posture-recognition-based MVFT may provide additional short-term benefits for improving neglect symptoms, balance function, and activities of daily living in patients with poststroke neglect. However, given the retrospective design and relatively short intervention period, larger prospective randomized controlled studies with long-term follow-up are required before routine clinical implementation can be recommended.

## Author contributions

**Conceptualization:** Juan Jin, Hongji An, Juanxiu Cui, Long Piao.

**Data curation:** Juan Jin, Hongji An, Juanxiu Cui, Long Piao.

**Formal analysis:** Juan Jin, Hongji An, Juanxiu Cui, Long Piao.

**Funding acquisition:** Juan Jin.

**Investigation:** Juan Jin.

**Writing – original draft:** Juan Jin.

**Writing – review & editing:** Juan Jin.
